# The plasma D-dimer trends and their value in acute lower limb ischemia patients treated by catheter directed thrombolysis

**DOI:** 10.1038/s41598-021-89905-x

**Published:** 2021-05-17

**Authors:** Xiaochun Liu, Hailiang Xie, Guofu Zheng, Yuanfei Liu

**Affiliations:** 1 The Department of General Surgery, Ganzhou People’s Hospital, Ganzhou, 341000 Jiangxi People’s Republic of China; 2 The Department of Intensive Care Unit, Ganzhou People’s Hospital, No. 17, Red flag avenue, Ganzhou, 341000 Jiangxi People’s Republic of China

**Keywords:** Embolism, Peripheral vascular disease, Thromboembolism, Thrombosis

## Abstract

To investigate the change trends of plasma D-dimer during catheter-directed thrombolysis (CDT) in acute lower limb ischemia (ALI) patients and their clinical value. A retrospective review of patients with ALI who received CDT was carried out. The repeated measurements of plasma D-dimer were analyzed by generalized estimating equations (GEEs) and the change trends of D-dimer were analyzed by spline regression approach. A total of 150 patients were included. Among them, 3 days of CDT was ineffective in 41 cases, effective in 33 cases and markedly effective in 76 cases. The results of GEEs analysis showed that serum D-dimer changed significantly with time (time effect, P < 0.001). Serum D-dimer levels of patients with different treatment outcomes were different after treatment (group effect, P < 0.001), and serum D-dimer levels in these three groups showed different trends over time (group*time effect, P < 0.001). The different trends in serum D-dimer level with time after treatment in the three groups could be directly seen in the spline regression curve (P < 0.001). The plasma D-dimer changes regularly during CDT for ALI. We can predict the efficacy of CDT and guide adjustments of the therapeutic regimen according to the trend of D-dimer changes during thrombolysis.

## Introduction

Acute lower limb ischemia (ALI) is caused by a reduction in arterial perfusion in the limb due to embolic migration or local thrombosis. In addition to the risk of limb damage or loss, it can cause life-threatening complications related to metabolic conditions triggered throughout the body. ALI should be treated urgently^1^. Catheter-directed thrombolysis (CDT) has been used for the treatment of ALI for over 20 years^2–4^. Thrombolytic therapy can cause significant changes in plasma D-dimer^5–7^. D-dimer is a soluble fibrin degradation product that is the result of the orderly breakdown of thrombi by the fibrinolysis system. Its level is commonly used in the clinical diagnosis and treatment evaluation of venous thromboembolic (VTE)^8^. Thrombolytic efficacy can be evaluated by monitoring plasma D-dimer levels during CDT in patients with deep vein thrombosis^9,10^. Before and after thrombolysis, the changes in plasma D-dimer levels are also considered to be significantly correlated with coronary recanalization, which could be used to predict the thrombolysis efficacy after acute myocardial infarction^6,7^.

The correlation between plasma D-dimer level and reperfusion has not yet been clarified in CDT applied for patients with ALI. Therefore, the purpose of this study was to investigate the correlation between the change trends of plasma D-dimer levels before and after CDT in ALI and lower limb arterial recanalization and whether the plasma D-dimer levels could predict the efficacy of thrombolysis.

## Methods

### Ethics and consent statement

This retrospective cohort study was approved by the Medical Ethics Committee of Ganzhou People’s Hospital, and the experiments were carried out in accordance with the approved guidelines. For this retrospective study, informed consent was waived by the Medical Ethics Committee of Ganzhou People’s Hospital.

### Study population

We retrospectively analyzed consecutive patients with ALI treated with CDT at Ganzhou People's Hospital from January 2013 to December 2019. Inclusion criteria: Patients receiving CDT intravascular treatment for ALI who were treated for at least 3 days. The exclusion criteria were as follows: (1) nonacute lower limb ischemia; (2) incomplete data; (3) patients receiving mechanical thromboembolism treatment; and (4) CDC duration shorter than 3 days.

The data of interest were collected through the electronic medical record system, focusing on plasma D-dimer levels during thrombolysis. In addition, according to the research needs, we collected the basic information of the eligible patients, including gender, age, alcohol consumption, smoking, degree of ischemia (Rutherford classification^11^), artery occlusion site, length of the thrombolytic catheter, and urokinase usage. Comorbidities of the patients were also collected, including hypertension, diabetes mellitus, atrial fibrillation, ischemic heart disease, cerebrovascular disease, renal insufficiency, chronic obstructive pulmonary disease, hyperlipidemia, and liver function.

### CDT procedure

The CDT procedure involves placing a multihole infusion catheter (Unifuse, AngioDynamics) into the thrombosis occlusion arterial segment of the lower limb under the guidance of digital subtraction angiography (DSA) and continuous infusion urokinase through the catheter. Urokinase was used at a dose of 30,000 to 50,000 U/h and then adjusted in accordance with the plasma fibrinogen levels. Thrombolysis would be stopped if the thrombus was completely dissolved or if the patient's plasma fibrin level was < 1 g/L or complications of severe hemorrhage (such as bleeding at the puncture site or other sites) occurred after thrombolysis.

### Plasma D-dimer level monitoring

Blood samples to measure circulating levels of plasma D-dimer were collected from the peripheral blood on admission, and serial samples were then collected at 8, 16, 24, 32, 40, 48, 56, 64, and 72 h post-CDT. At least 10 repeated measurements of plasma D-dimer were performed in each case.

### Grouping

For the purpose of this analysis, the patients were further divided into three subgroups depending on the immediate imaging results of the DSA images taken on the third day of CDT and limb distal arterial pulsation and patient symptom improvement.

#### Markedly effective group

DSA images showed occluded segment fully open; pulsation of the dorsal foot artery and/or posterior tibial artery were palpable; and the lower extremity pain completely disappeared.

#### Effective group

DSA images showed that the occluded segment was completely or partially unobstructed. The dorsal foot artery and/or posterior tibial artery pulses were not detectable, but the skin temperature of the lower extremity was warm, and the pain was relieved.

#### Ineffective group

DSA images showed the occluded segment was still obstructed; the dorsal foot artery and/or posterior tibial artery pulses were not palpable; and the lower extremity pain did not improve.

### Statistical analysis

SPSS software package version 24.0 (IBM, Armonk, NY, USA) and the R Programming Language (version 4.0.3) was used for data management and statistical analysis. The nonparametric data were expressed as medians (interquartile ranges, IQR), and categorical data were presented as percentages. The ordinal data for the three groups was analyed by Kruskal Wallis Test. The data of repeated measurements of plasma D-dimer obtained from the three groups in the research process were longitudinal data, and each data was not independent, and its intra-group correlation could not be ignored. In order to get a more accurate conclusion, the effect of thrombolysis in the three groups was statistically analyzed by the statistical approach of generalized estimating equations (GEEs) base on gamma distribution.

Spline regression approach was used to analyze the change trends in the D-dimer levels among the three groups by R Programming Language. All tests were two-sided with a significance level of 0.05.

## Results

### General data of the patients

**Figure 1:**
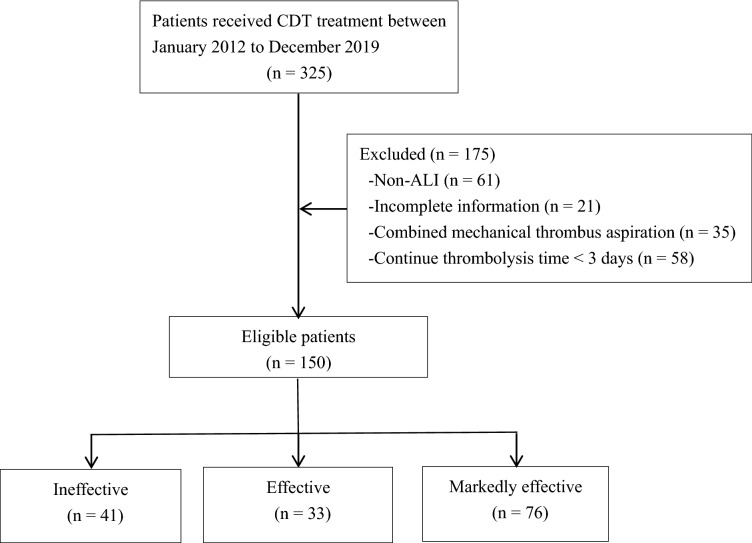
Patient flowchart.

From January 2013 to December 2019, 325 patients with lower limb ischemia received CDT treatment in our hospital. Of them, 61 patients had non-ALI, 21 patients had incomplete information (no repeated measurement data of D-dimer), 35 patients underwent mechanical thrombus aspiration at the same time, and 58 patients continued thrombolysis for < 3 days (patients who had the thrombolytic catheter removed early because they had achieved good thrombolytic results or were unable to adhere to CDT due to complications such as severe bleeding), so the remaining 150 patients who met the inclusion criteria were included in the analysis (Fig. 1).

The average age was 76.1 (56–79) years, and 81 were males. The ischemic level of Rutherford IIb accounted for the highest proportion (92 cases, 61.3%) and the average duration of symptoms was 1.5 days. The most common site of occlusion was the femoral popliteal artery (62 cases, 41.3%). Three days of CDT was ineffective in 41 cases (27.3%) and effective in 33 cases and markedly effective in 76 cases, accounting for 73.7%. Among them, a total of 5 patients (3.4%) required amputation, including 1 major amputation and 4 minor amputations. The patients were divided into three groups according to their outcome, and the baseline characteristics of the three groups are shown in Table 1. There were no statistically significant differences among the three groups for the degree of ischemia, occlusion site, type of thrombolytic catheter or amount of urokinase. Eleven patients in the ineffective group were treated with CDT for more than 3 days.

**Table 1:** Baseline characteristics of the patients.

Variable	Total, (n = 150)	Ineffective (n = 41)	Effective (n = 33)	Markedly effective (n = 76)	P value^a^
**Gender**					
Male, No. (%)	81 (54.0)	20 (48.8)	22 (66.7)	39 (51.3)	0.249
Age, median (IQR) (years)	67.1 (56–79)	64.37 (47–81)	67.88 (59–78)	68.24 (56–79)	0.743
Smoking,	62 (41.3)	14 (34.1)	13 (39.4)	35 (46.1)	0.447
Alcohol consumption	26 (17.3)	9 (22.0)	2 (6.1)	15 (19.7)	0.148
**Comorbidities**					
Hypertension	72 (48.0)	16 (39.0)	16 (48.5)	40 (52.6)	0.374
Diabetes mellitus	36 (24.0)	7 (17.1)	7 (21.2)	22 (28.9)	0.329
Atrial fibrillation	60 (40.0)	19 (46.3)	15 (45.5)	26 (34.2)	0.342
Ischemic heart disease	41 (27.3)	9 (22.0)	13 (39.4)	19 (25.0)	0.202
Cerebrovascular disease	30 (20.0)	5 (12.2)	7 (21.2)	18 (23.7)	0.329
Renal insufficiency	67 (44.7)	15 (36.6)	17 (51.5)	35 (46.1)	0.415
Chronic obstructive pulmonary disease	34 (22.7)	7 (17.1)	10 (30.3)	17 (22.4)	0.402
Hyperlipidemia	70 (46.7)	22 (53.7)	15 (45.5)	33 (43.4)	0.566
**Liver function**^**b**^					
A	94 (62.7)	25 (61.0)	23 (69.7)	46 (60.5)	0.645
B	51 (34.0)	13 (31.7)	9 (27.3)	29 (38.2)	
C	5 (3.3)	3 (7.3)	1 (3.0)	1 (1.3)	
**Degree of ischemia**^**c**^					
I	16 (10.7)	4(2.7)	5 (3.3)	7 (4.7)	0.735
IIa	36(24.0)	9 (6.0)	7 (4.7)	20 (24.0)	
IIb	92 (61.3)	25 (16.7)	20 (13.3)	47 (31.3)	
III	6 (4.0)	3 (2.0)	1(0.7)	2 (1.3)	
**Artery occlusion site**					
Iliac artery	12 (8.0)	0 (0.0)	6 (18.2)	6 (7.9)	0.184
Superficial temporal artery	22 (14.7)	14 (34.1)	5 (15.2)	3 (3.9)	
Popliteal artery	38 (25.3)	9 (22.0)	4 (12.1)	25 (32.9)	
Iliac femoral artery	8 (5.3)	4 (9.8)	4 (12.1)	0 (0)	
Femoral popliteal artery	62 (41.3)	10 (24.4)	12 (36.4)	40 (52.6)	
Iliofemoral popliteal artery	3 (2.0)	3 (7.3)	0 (0.0)	0 (0)	
Tibiofibular artery	5 (3.3)	1 (2.4)	2 (6.1)	2 (2.6)	
**Length of the thrombolytic catheter**					
10 cm	34 (22.7)	4 (9.8)	3 (9.1)	27 (35.5)	0.216
20 cm	38 (25.3)	14 (34.1)	11 (33.3)	13 (34.2)	
30 cm	23 (15.3)	11 (26.8)	3 (9.1)	9 (11.8)	
40 cm	29 (19.3)	8 (19.5)	10 (30.3)	11 (14.5)	
50 cm	26 (17.3)	4 (9.8)	6 (18.2)	16 (21.1)	
**Amputation**					
Minor amputation	4 (2.7)	4 (9.8)	0 (0)	0 (0)	0.0001
Major amputation	1 (0.7)	1 (2.4)	0 (0)	0 (0)	
Urokinase usage median (IQR), (*10^4^u)	252.27 (230–280)	245.81 (230–260)	257.27 (240–280)	253.95 (220–290)	0.309

*IQR* interquartile range.

^a^Kruskal Wallis Test.

^b^Child-Pugh classification.

^c^Ruther classification.

### Plasma D-dimer levels of patients

The median (IQR) values of serum D-dimer changes in the three groups within 72 h after treatment are shown in Table 2. The results of GEEs analysis showed that serum D-dimer changed significantly with time (time effect, P < 0.001) within 72 h after treatment. Serum D-dimer levels of patients with different treatment outcomes in these three groups were different after treatment (group effect, P < 0.001), and after treatment, serum D-dimer levels in these three groups showed different trends over time (group*time effect, P < 0.001). Specifically, the serum D-dimer levels of the markedly effective group were higher than those of ineffective and effective groups at the 8- and 16-h time points (P < 0.05). At 48–72 h, the serum D-dimer levels of both the markedly effective and effective groups were higher than those of the ineffective group (P < 0.05).

**Table 2:** Plasma D-dimer levels of patients.

Time	Ineffective (n = 41)	Effective (n = 33)	Markedly effective (n = 76)
0 h	1.83 (1.17, 3.67)	1.23 (0.74, 2.54)	1.73 (1.01, 2.27)
8 h	9.32 (6.45, 16.29)	3.99 (3.22, 8.78)	13.57 (6.25, 46.76) ^ab^
16 h	29.34 (17.89, 38.98)	32.12 (5.83, 40.55)	127.3 (26.09, 228.44) ^ab^
24 h	19.03 (12.36, 67.41)	26.22 (4.22, 39.55)	49.88 (18.35, 83.91)
32 h	17.65 (10.01, 74.66)	17.81 (8.6, 67.92)	32.38 (16.91, 54.09)
40 h	24.94 (15.16, 49.12)	8.82 (5.57, 39.73)	12.32 (5.56, 26.31) ^a^
48 h	25.01 (19.48, 75.32)	20.54 (3.18, 33.43) ^a^	4.29 (3.11, 12.19) ^ab^
56 h	23.59 (13.43, 94.58)	8.99 (2.26, 23.04) ^a^	2.45 (1.76, 3.34) ^ab^
64 h	19.09 (9.00, 33.10)	2.19 (1.02, 6.18) ^a^	1.49 (1.07, 2.01) ^ab^
72 h	11.75 (4.78, 13.50)	1.23 (0.78, 2.67) ^a^	0.72 (0.60, 0.99) ^ab^
Time effect	Wald χ^2^ = 2458.61, df = 9, p < 0.001		
Group effect	Wald χ^2^ = 43.68, df = 2, p < 0.001		
Group* time effect	Wald χ^2^ = 1580.32, df = 2, p < 0.001		

Data expressed as median (IQR). Generalized estimating equations models base on gamma distribution were performed.

^a^*p* < 0.05 compared with ineffective group at the same time point.

^b^*p* < 0.05 compared with effective group at the same time point.

**Figure 2:**
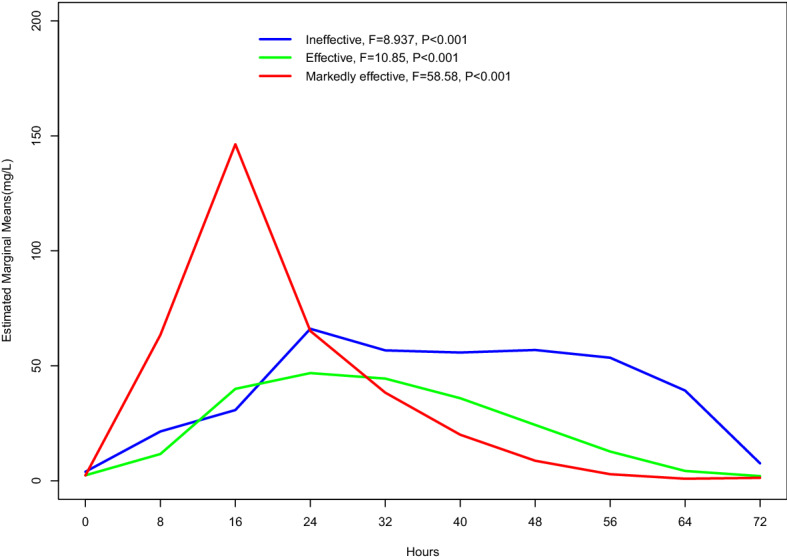
Spline regression curves for the repeated measurements of D-dimer.

The different trends in serum D-dimer level with time after treatment in the three groups could also be directly seen in the spline regression curve after fitting successfully (Fig. 2, P < 0.001). The trend chart showed that the plasma D-dimer level in all three groups increased at the beginning and then decreased after thrombolysis. The D-dimer level in the markedly effective group increased rapidly at the beginning of thrombolysis, reached its peak 16 h later, and then decreased quickly to the preoperative level. The D-dimer of the ineffective and effective groups increased slowly after beginning thrombolysis, peaked 24 h later, and then slowly declined. In the ineffective group, the plasma D-dimer level increased again 48 h after the beginning of thrombolysis and had another small peak.

## Discussion

By analyzing the plasma D-dimer change trends, we can predict the reperfusion of ischemic lower limbs after thrombolytic therapy and know the results of CDT in a timely manner. This has rarely been reported in previous studies.

ALI is caused by a sudden interruption of the main blood perfusion of the lower limb for various reasons, and it usually requires immediate revascularization. CDT has become one of the most commonly used methods for the treatment of ALI^12,13^. After thrombolytic therapy, the vascular stenosis, occlusion and other lesions can be treated simultaneously with interventional therapy, which improves the short-term and long-term patency rate of such occlusive lesions and reduces the need for open surgery in most of these patients^4^.

D-dimer is the degradation product of cross-linked fibrin, and its plasma level reflects the formation and degradation of fibrin. D-dimer is considered a useful biomarker because of its potential to identify individuals in a high coagulation state. D-dimer is often associated with thrombi, and its elevation should be of continuing concern^14–16^. D-dimer has been widely used in the diagnosis of VTE^8,17,18^. There have also been many relevant studies in some cardiovascular diseases, such as atrial fibrillation^19–21^, coronary heart disease^14,22,23^, stroke^24,25^ and aortic dissection^26–28^, and D-dimer can even be used as a predictor of the diagnosis and prognosis of such diseases^29–32^.

D-dimer levels are lower in the circulation in healthy individuals and higher in the presence of thrombi^8^. During the CDT process, the fibrin in the thrombus degrades, and D-dimer is produced. These dissolved substances are continuously released into the blood, causing the plasma D-dimer content to change constantly^33,34^. The D-dimer level increases continuously during thrombolysis and is positively correlated with the degree of thrombolysis^5^. D-dimer has a half-life of approximately 8 h until it is cleared by the kidney and reticuloendothelial system^35^. In the later stage of thrombolysis, with the gradual opening of the occluded vessels, the thrombus load in the vessels decreases, and the D-dimer content in the blood decreases continuously until it returns to the prethrombolysis level. Monitoring the changes in plasma D-dimer content pre- and posttreatment is also often used to evaluate the therapeutic effect^7,9,33,34^. Cakar, M. A. et al. analyzed D-dimer levels pre- and post-thrombolysis in 186 patients with acute coronary syndrome treated with intravenous tissue-type plasminogen activator (100 mg) or streptokinase (1,500,000 U) and found that D-dimer levels were markedly high after thrombolytic therapy versus before (155 mg/dl, 362 mg/dl, P < 0.005)^6^.

For the convenience of analysis, we grouped our patients according to the results of thrombolysis. The change trends of D-dimer in the three groups during thrombolysis are shown in Fig. 2. During thrombolytic therapy, the lower limb arteries of patients whose plasma D-dimer level rose rapidly, reached its peak, and then rapidly dropped to the preoperative level obtained complete recanalization. The lower limb arteries of patients whose plasma D-dimer level rose slowly, then reached its peak slowly or had two peaks, and then dropped slowly to the preoperative level might achieve partial recanalization or remain occluded.

Accordingly, we believe that after CDT treatment of ALI, if the plasma D-dimer levels show a significant increase within a short period of time and then a rapid decline without a rebound, thrombolytic therapy is markedly effective. If the plasma D-dimer continues to rise slowly and decline slowly, this suggests that the thrombolytic catheter may not be sufficient contact with the thrombus or that the thrombolytic drug dose is insufficient, which requires appropriate adjustments. If the D-dimer exhibits multiple peaks, this may be due to an obstructed vascular outflow tract or occlusion of the artery by the old thrombus, which should be resolved by combining CDT with other treatment methods^36^. In the process of CDT, no change or a weak change in the monitored D-dimer level often indicates the presence of an arteriosclerotic plaque-occluded artery in the patient. Therefore, it is necessary to improve the blood supply of the ischaemic extremity as soon as possible by combining CDT with percutaneous transluminal angiography (PTA)/stent implantation^36,37^ rather than waiting for 3 days until the completion of CDT. There were 58 patients whose CDT process did not reach 3 days during the study period. In addition, the D-dimer level of some patients continued to increase and slowly decreased during the CDT process, indicating a large amount of thrombus or the presence of a new thrombus, and it was necessary to extend the CDT time or adjust the location and length of the thrombolytic catheter. The CDT duration was longer than 3 days in 11 patients during our study period. Of course, if inevitable ischaemic necrosis of the affected limb occurs during the CDT process, the CDT process should be terminated in time for amputation, rather than blindly forcing the completion of unnecessary thrombolytic therapy. Five of the patients in the ineffective group underwent limb amputation for inevitable limb necrosis.

Several limitations of this analysis should be acknowledged. In this study, patients with CDT thrombolysis for 1–2 days were excluded because these patients did not have 3 days of complete D-dimer data, which reduced the sample size of our study. In addition, the grouping in this study was based on the recovery of the lower limb blood supply of patients on the third day of CDT. In fact, patients with a poor CDT response were still receiving treatment after 3 days, such as continued CDT treatment or a combination of other treatments, so the final clinical results for all of the patients were not provided in this study. In addition, CDT therapy for non-ALI is also widely used in clinical practice^38,39^, but the lesions in these patients are not mainly thrombotic and often require PTA and/or stent implantation, so they were excluded from this study. Therefore, a multicenter, prospective, randomized controlled study may be the best way to further understand the trends and clinical significance of plasma D-dimer changes during CDT in patients with ALI (Supplementary Information S1).

## Conclusion

In conclusion, the plasma D-dimer level changes regularly during CDT for ALI. Occluded arteries may be recanalized in patients whose D-dimer appears to rise rapidly during CDT, peaks, and then rapidly declines to pretreatment levels. Therefore, we can predict the results of CDT and guide adjustment of the therapeutic regimen according to the trends of D-dimer changes during thrombolysis to obtain better clinical effects and allow more ischemic limbs to be salvaged.

## Supplementary Information

Supplementary Information.

## References

[1] Creager MA, Kaufman JA, Conte MS (2012). Acute limb ischemia. N. Engl. J. Med..

[2] Theodoridis PG (2018). Thrombolysis in acute lower limb ischemia: Review of the current literature. Ann. Vasc. Surg..

[3] Ouriel, K., Veith, F. J. & Sasahara, A. A. A comparison of recombinant urokinase with vascular surgery as initial treatment for acute arterial occlusion of the legs. Thrombolysis or Peripheral Arterial Surgery (TOPAS) Investigators. *N Engl J Med* **338**, 1105–1111. 10.1056/NEJM199804163381603 (1998). 10.1056/NEJM199804163381603 9545358

[4] Grip O, Wanhainen A, Acosta S, Bjorck M (2017). Long-term outcome after thrombolysis for acute lower limb ischaemia. Eur. J. Vasc. Endovasc. Surg..

[5] Liu M, Zhang F (2018). Administration routes affect thrombolytic effect of catheter-directed thrombolysis with pro-urokinase in treating deep vein thrombosis. Ann. Transl. Med..

[6] Cakar MA (2013). Correlation between D-dimer levels and coronary artery reperfusion in acute myocardial infarction patients after thrombolytic treatment. Blood Coagul. Fibrinolysis.

[7] Brenner B (1989). Relation of plasma D-dimer concentrations to coronary artery reperfusion before and after thrombolytic treatment in patients with acute myocardial infarction. Am. J. Cardiol..

[8] Weitz JI, Fredenburgh JC, Eikelboom JW (2017). A test in context: D-dimer. J. Am. Coll. Cardiol..

[9] Engelberger RP (2019). Enhanced thrombolysis by ultrasound-assisted catheter-directed thrombolysis and microbubbles in an in vitro model of iliofemoral deep vein thrombosis. Thromb. Haemost..

[10] Gao G, Zhu S, Xie Z, Wang J, Yao B (2019). Efficacy of catheter-directed thrombolysis on post-burn deep venous thrombosis of lower extremity. J. Coll. Phys. Surg. Pak..

[11] Rutherford RB (2009). Clinical staging of acute limb ischemia as the basis for choice of revascularization method: When and how to intervene. Semin. Vasc. Surg..

[12] Lian WS (2020). Efficacy of intra-arterial catheter-directed thrombolysis for popliteal and infrapopliteal acute limb ischemia. J. Vasc. Surg..

[13] Grip O (2014). Outcome and complications after intra-arterial thrombolysis for lower limb ischaemia with or without continuous heparin infusion. Br. J. Surg..

[14] Gong P (2016). Plasma d-dimer as a useful marker predicts severity of atherosclerotic lesion and short-term outcome in patients with coronary artery disease. Clin. Appl. Thromb. Hemost..

[15] Narang J, Nowacki AS, Seballos SS, Wang PR, Mace SE (2020). D-dimer can help differentiate suspected pulmonary embolism patients that require anti-coagulation. Am. J. Emerg. Med..

[16] Yucel O, Yucel H, Zorlu A (2017). D-dimer is a predictor of cardiovascular death, and new-onset atrial fibrillation in patients with systolic heart failure. Int. J. Cardiol..

[17] Tang N (2017). Combined measurement of factor XIII and D-dimer is helpful for differential diagnosis in patients with suspected pulmonary embolism. Clin. Chem. Lab. Med..

[18] Cohen AT (2014). D-dimer as a predictor of venous thromboembolism in acutely ill, hospitalized patients: A subanalysis of the randomized controlled MAGELLAN trial. J. Thromb. Haemost..

[19] Siegbahn A (2016). D-dimer and factor VIIa in atrial fibrillation: Prognostic values for cardiovascular events and effects of anticoagulation therapy A RE-LY substudy. Thromb. Haemost..

[20] Hijazi Z, Oldgren J, Siegbahn A, Wallentin L (2017). Application of biomarkers for risk stratification in patients with atrial fibrillation. Clin. Chem..

[21] Ruff CT (2016). Cardiovascular biomarker score and clinical outcomes in patients with atrial fibrillation: A subanalysis of the ENGAGE AF-TIMI 48 randomized clinical trial. JAMA Cardiol.

[22] Simes J (2018). D-dimer predicts long-term cause-specific mortality, cardiovascular events, and cancer in patients with stable coronary heart disease: LIPID Study. Circulation.

[23] Folsom AR (2009). Associations of factor VIIIc, D-dimer, and plasmin-antiplasmin with incident cardiovascular disease and all-cause mortality. Am. J. Hematol..

[24] Folsom AR, Gottesman RF, Appiah D, Shahar E, Mosley TH (2016). Plasma d-dimer and incident ischemic stroke and coronary heart disease: The atherosclerosis risk in communities study. Stroke.

[25] Wang Y (2016). The correlation of D-dimer levels with patient outcomes in acute ischemic cerebrovascular disease complicating coronary heart disease. Neurol. Res..

[26] Watanabe H (2016). Diagnostic test accuracy of D-dimer for acute aortic syndrome: systematic review and meta-analysis of 22 studies with 5000 subjects. Sci. Rep..

[27] Asha SE, Miers JW (2015). A systematic review and meta-analysis of D-dimer as a rule-out test for suspected acute aortic dissection. Ann. Emerg. Med..

[28] Yuan SM, Shi YH, Wang JJ, Lu FQ, Gao S (2011). Elevated plasma D-dimer and hypersensitive C-reactive protein levels may indicate aortic disorders. Rev. Bras. Circ. Cardiovasc..

[29] Wang J, Wu XY, Liang Y, Guo W (2020). Predictive value of the Wells score combined with D-dimer level in identifying acute pulmonary embolism in patients with coronary heart disease with chest pain. Chin. Med. J. (Engl).

[30] Mjelva OR (2016). Long-term prognostic utility of pentraxin 3 and D-dimer as compared to high-sensitivity C-reactive protein and B-type natriuretic peptide in suspected acute coronary syndrome. Eur. J. Prev. Cardiol..

[31] Zhang X, Wang S, Sun L, Fang S, Yu B (2020). Prognostic value of D-dimer in acute myocardial infarction complicated by heart failure with preserved ejection fraction. ESC Heart Fail..

[32] Kremers B (2020). Plasma biomarkers to predict cardiovascular outcome in patients with peripheral artery disease: A systematic review and meta-analysis. Arterioscler. Thromb. Vasc. Biol..

[33] Takamura TA (2017). Circulating malondialdehyde-modified low-density lipoprotein (MDA-LDL) as a novel predictor of clinical outcome after endovascular therapy in patients with peripheral artery disease (PAD). Atherosclerosis.

[34] Hsu PJ (2016). High plasma D-dimer indicates unfavorable outcome of acute ischemic stroke patients receiving intravenous thrombolysis. Cerebrovasc. Dis..

[35] Hager K, Platt D (1995). Fibrin degeneration product concentrations (D-dimers) in the course of ageing. Gerontology.

[36] Schrijver AM (2015). Dutch randomized trial comparing standard catheter-directed thrombolysis and ultrasound-accelerated thrombolysis for arterial thromboembolic infrainguinal disease (DUET). J. Endovasc. Ther..

[37] George EL (2020). Real-World Outcomes of EKOS ultrasound-enhanced catheter-directed thrombolysis for acute limb ischemia. Ann. Vasc. Surg..

[38] Lukasiewicz A, Flisinski P, Lichota W (2020). Catheter-directed thrombolysis is not limited to acute limb ischemia treatment: experience from a division of vascular surgery. J. Cardiovasc. Surg. (Torino).

[39] Gunes Y, Sincer I, Erdal E (2019). Catheter-directed intra-arterial thrombolysis for lower extremity arterial occlusions. Anatol. J. Cardiol..

